# The Life After Stroke In Northern Sweden Study (LASINS): Methodology, cohort demographics and initial results

**DOI:** 10.3233/NRE-230278

**Published:** 2024-03-11

**Authors:** Maria Kähler, Hanna M. Nilsson, Jan Lexell

**Affiliations:** a Department of Health Sciences, Rehabilitation Medicine Research Group, Lund University, Lund, Sweden; b Department of Rehabilitation, Sunderby Hospital, Luleå, Sweden; c Department of Rehabilitation, Ängelholm Hospital, Ängelholm, Sweden

**Keywords:** Depression, fatigue, fatigue assessment scale (FAS), international classification of functioning, disability and health, outcome assessment, health care, exercise, sleep wake disorders, stroke, sleep

## Abstract

**BACKGROUND:**

To advance rehabilitation we need a comprehensive understanding of functioning and disability of people after stroke.

**OBJECTIVE:**

To present an overview of the methodology of the Life After Stroke In Northern Sweden Study, compare participants and non-participants regarding gender and age, and describe baseline sociodemographics, stroke characteristics and the participants’ self-rated degree of recovery.

**METHODS:**

Data were collected through a study specific questionnaire, from the participants’ medical records and with internationally established self-assessment tools focusing on sleep disturbances, depressive symptoms, fatigue, physical activity, and remaining physical and cognitive impairments, activity limitations, participation restrictions and life satisfaction.

**RESULTS:**

Of 301 potential participants, 160 comprise the final sample (response rate 53%; 86 men and 74 women, mean age 73 years±11, mean time since stroke onset 35 months±11; 18– 61). Most participants had an ischemic stroke (87%), were retired (84%), cohabitant (63%) and walked independently (71%). The mean self-rated degree of recovery was 75 (SD±24; 0– 100).

**CONCLUSIONS:**

These baseline data together with forthcoming studies will describe stroke-related impairments, activity limitations, participation restrictions and life satisfaction more than one year after stroke, and deepen our understanding of factors of importance for a healthy and successful life after stroke.

## 1 Introduction

Stroke is one of the most common causes of death globally (Lopez et al., 2006; Vos et al., 2020). It is also the disease in the adult population that most commonly leads to a significant disability (GBD 2016 Neurology Collaborators, 2019; World Health Organization [WHO], 2011). Despite improvements in treatment many people suffer from remaining motor, sensory and cognitive impairments after stroke. These impairments can affect their daily activities with a need for rehabilitation to cope with challenges in everyday life (WHO, 2011). It is also well known that people after stroke can have an inactive lifestyle and experience participation restrictions, and thereby reduced life satisfaction (Byun et al., 2020; Donkor, 2018; Faria-Fortini et al., 2016). Thus, studies assessing stroke survivors functioning and disability are needed to advance management, rehabilitation and long-term follow-up.

One of the remaining impairments that people can experience after stroke is sleep disturbances (Iddagoda et al., 2019). Up to two thirds of stroke survivors report sleep disturbances, which is significantly higher than in the non-disabled population (Baylan et al., 2020). Sleep disturbances after stroke is also associated with depressive symptoms (Davis et al., 2019). Up to one third of stroke survivors show depressive symptoms, regardless of follow-up time and this, in turn, is associated with negative rehabilitation outcomes (Hackett et al., 2005; Medeiros et al., 2020). Another common impairment after stroke is fatigue. As many as 62% of stroke survivors report fatigue and fatigue is also common in people with limited or no physical impairments (Cumming et al., 2016). The severity of fatigue after stroke is often high (Glader et al., 2002) and in up to 40% of stroke survivors it is one of the most pronounced impairments (Ingles et al., 1999).

These common stroke-related impairments, together with reduced physical performance, can lead to an inactive lifestyle, which can further affect a person’s ability to function in daily life (Alghwiri, 2016; Izquierdo et al., 2021). On one hand, many stroke survivors do not achieve the recommended amount of physical activity according to the WHO (WHO, 2010; Bull et al., 2020; Fini et al., 2017). On the other hand, it has been reported that an increased physical activity after a stroke could improve recovery (Cook, 2020) and quality of life (Belfiore, 2018), reduce disability (Saunders et al., 2016) and improve cognitive function (Kim et al., 2019), and potentially affect sleep, depressive symptoms and fatigue.

Despite increased attention to research on functioning and disability in people after stroke, many questions are still unanswered. Studies of remaining impairments and physical activity have generally been performed in the early phase, within the first year after stroke onset, hence there is a dearth of information in a more long-term perspective. Moreover, our understanding of the associations and interactions between remaining impairments and physical activity, activity limitations, participation restrictions and life satisfaction in stroke survivors is incomplete. Because of cultural and contextual differences, it can be challenging to relate findings across studies and between different national contexts. Therefore, further studies and from different parts of the world are needed to provide a comprehensive understanding of functioning and disability among people after a stroke.

To contribute to our understanding of the life situation among stroke survivors, we initiated the Life After Stroke In Northern Sweden Study (LASINS). The LASINS is a cross-sectional, population-based survey with a neurorehabilitation approach aiming to provide an in-depth understanding of factors of importance for a healthy and successful life at least one year after stroke onset. In the LASINS, we focus on sleep disturbances, depressive symptoms, fatigue and physical activity, and the interaction with remaining physical and cognitive impairments, activity limitations, participation restrictions and life satisfaction.

The objectives of the present study are to: (i) present an overview of the methodology (i.e., research design, recruitment procedure, data collection and assessment tools) of the LASINS, (ii) compare participants and non-participants regarding gender and age, and (iii) describe baseline sociodemographics, stroke characteristics and the participants’ self-rated degree of recovery.

## 2 Materials and methods

### 2.1 Study design

The LASINS has a combined quantitative exploratory and descriptive design targeting adult stroke survivors at least one year after stroke onset. Follow-up studies are planned to allow for a longitudinal approach together with qualitative studies to explore outcome from a truly person-centered perspective.

The participants are resident in Norrbotten County, the most northern region of Sweden. Norrbotten County corresponds to a quarter of the area of Sweden and with a current population of about 250 000 people, representing 2.5% of Sweden’s population. In Sweden, approximately 25 000 people suffer from a stroke every year, of which around 600 live in Norrbotten County (Riksstroke, 2022a; The Swedish National Board of Health and Welfare, 2022).

The LASINS was designed, and the self-assessment tools selected, based on the International Classification of Functioning, Disability and Health (ICF) (WHO, 2023). The ICF has a biopsychosocial approach to disabilities, and provides a structured framework and a common language to describe functioning and disability in relation to health (Lexell & Brogårdh, 2015). ICF also provides a scientific basis to understand and study health with regard to body functions and structures as well as activities and participation (WHO, 2023).

The LASINS follows the Strengthening the Reporting of Observational studies in Epidemiology guidelines (STROBE) and contains the necessary items according to the STROBE checklist (Equator Network, 2023; Strobe, 2023) to properly report an observational study (von Elm et al., 2007).

### 2.2 Ethical considerations

The principles of the Declaration of Helsinki on research involving humans are followed. All participants were informed about the study and that they could withdraw at any time. Written informed consent was obtained from all participants. The LASINS was approved by the Swedish Ethical Review Authority (number: 2021-01408; date: April 25, 2021).

### 2.3 Study population

All persons that had been diagnosed with a stroke (ICD-10: I61 (cerebral hemorrhage); I63 (cerebral infarction); I64 (acute cerebrovascular disease not specified as hemorrhage or infarction)) and admitted to the stroke unit at a regional hospital in Norrbotten County between January 2017 and December 2019 were included. Further inclusion criteria were: living in ordinary housing, aged 18 years and older, and being able to understand and answer a questionnaire and self-assessment tools in Swedish. Exclusion criteria were: a transient ischemic attack (TIA), severe cognitive impairment, not speaking/understanding Swedish and having moved abroad.

**Fig. 1:**
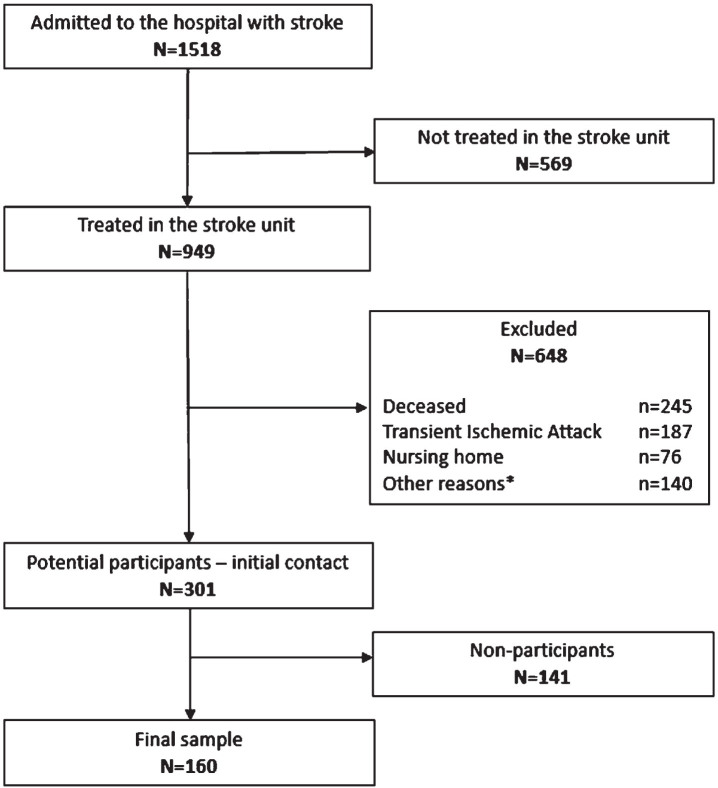
The recruitment procedure of participants in the Life After Stroke In Northern Sweden Study (LASINS). *Other reasons included: severe cognitive impairment and dementia, not speaking/understanding Swedish, and moved abroad.

Based on the inclusion and exclusion criteria, potential participants were identified through reviews of the medical records (Fig. 1). A total of 1 518 people had been admitted with a stroke to the hospital of which 949 persons (63%) had been admitted to the stroke unit. Of these, 301 people met the inclusion criteria and were invited to participate (a majority of those excluded were decesed). A total of 160 people accepted the invitation and thereby comprise the final sample (response rate 53%).

### 2.4 Data collection procedures

Data collection was conducted by two of the authors (MK and HMN) between June 2021 and February 2022. The authors mailed information about the study to the potential participants (N = 301), an informed consent form, a study specific questionnaire with sociodemographic data, and the self-assessment tools. A pre-paid envelope was enclosed to return the informed consent form, the questionnaire and the self-assessment tools. One reminder was sent to those who had not responded after the first invitation.

### 2.5 Data obtained from sociodemographic questionnaire and medical records

The following sociodemographic data were obtained from the questionnaire: gender, age, marital status, vocational situation, use of mobility device and need of home help. Data collected from the participants’ medical records were: time since stroke onset, type of stroke, location of stroke, stroke treatment, first time stroke and co-morbidities.

### 2.6 Self-assessment tools

The Swedish versions of six self-assessment tools (five generic and one stroke specific) were chosen after literature searches, from previously performed stroke research, and the authors clinical experience and experiences from rehabilitation outcomes research. In Table 1, the self-assessment tools are presented according to the components of the ICF (Body functions and structures, activities and participation) (WHO, 2023) that they are related to.

**Table 1:** The assessment tools used in the Life After Stroke In Northern Sweden Study (LASINS) and their relationship to the International Classification of Functioning, Disability and Health (ICF)

ICF Part	Functioning and Disability		
ICF Component	Body Functions & Structures	Activity	Participation
Generic assessment tools
Fatigue Assessment Scale (FAS)	X
Geriatric Depression Scale (GDS-20)	X
Life Satisfaction Questionnaire (LiSat-11)			X
Pittsburgh Sleep Quality Index (PSQI)	X
Swedish National Board of Health and Welfare Physical Activity questionnaire (BHW PA)		X
Stroke specific assessment tool
Stroke Impact Scale (SIS 3.0)	X	X	X

#### 2.6.1 Fatigue Assessment Scale (FAS)

The FAS was originally developed to assess fatigue in people with sarcoidosis (De Vries et al., 2004) and has subsequently been used in other diagnostic groups. The FAS comprises ten questions about a person’s general feelings and uses a 5-point rating scale from one (never) to five (always). The maximum score is 50 where a greater score reflects more fatigue; a score of 24 or more is suggested to indicate fatigue in the stroke population (Cumming et al., 2017; Michielsen et al., 2003). The Swedish version of the FAS has been tested on people after stroke and has shown good internal consistency (Cronbach’s alfa 0.82), good reliability with an Intraclass Correlation Coefficient (ICC) of 0.73 and good validity compared with other assessment tools (*r* = – 0.73 and 0.62) (Bråndal et al., 2016). The FAS can also distinguish between fatigue and depression (De Vries et al., 2004). The FAS is used with permission according to ©FAS (Fatigue Assessment Scale): ild care foundation (www.ildcare.nl).

#### 2.6.2 Geriatric Depression Scale (GDS-20)

The GDS is a screening tool for depression in older adults and the original tool includes 30 questions with response options ‘yes’ or ‘no’. GDS-30 has been shown to be reliable, has a high degree of internal consistency (Cronbach’s alpha 0.94) and good validity compared with other assessment tools (*r* = 0.83 and 0.84) (Yesavage et al., 1982). It has also been tested on people after stroke and shown to be valid and reliable (Agrell et al., 1989). The original GDS has subsequently been shortened to 15 questions and in the Swedish version five questions have been added, yielding 20 questions with a total score of 20 (i.e., GDS-20); if the score is 6 or more depression should be suspected. GDS-20 is also considered reliable as it distinguishes depressed persons from non-depressed in a consistent way and has shown good validity when compared to other assessment tools (*r* = 0.61) (Gottfries et al. 1997).

#### 2.6.3 Life Satisfaction questionnaire (LiSat-11)

LiSat-11 was originally developed and validated by Fugl-Meyer et al. (Fugl-Meyer et al., 1991; Fugl-Meyer et al., 2002). LiSat-11 comprises one global item (Life as a whole) and ten domain-specific items (vocation, economy, leisure, contacts with friends, sexual life, activities of daily living (ADL), family life, partner relationship, somatic health and psychological health). There are 6 response options to each item ranging from 1 (very dissatisfied) to 6 (very satisfied). The response options can also be dichotomized as satisfied (score 5 and 6) and dissatisfied (score 1 to 4). Lisat-11 has a high degree of internal consistency with a Cronbach’s alpha of 0.85 (Fugl-Meyer et al., 2002). The test-retest reliability in people after stroke has been shown to be high for all eleven items with a Kappa coefficient from 0.59 to 0.97 (Ekstrand et al., 2018a).

#### 2.6.4 Pittsburgh Sleep Quality Index (PSQI)

The PSQI was developed to assess sleep quality and distinguish people with good sleep from those with sleep disturbances (Buysse et al., 1989). The PSQI includes seven different components of sleep: subjective sleep quality, sleep latency, sleep duration, habitual sleep efficiency, sleep disturbances, use of sleeping medication and daytime dysfunction. The individual components have a value of 0 to 3 which are then summed to a global score with a maximum of 21; a greater score reflects more sleep disturbances and 5 or more is the cut-off value between good sleep quality and sleep disturbances (Buysse et al., 1989). The internal consistency has been shown to be good with Cronbach’s alpha from 0.70 to 0.83 (Buysse et al., 1989; Mollayeva et al., 2016). The PSQI has a correlation coefficient of 0.80 with other questionnaires and the test-retest reliability is high with an ICC from 0.70 to 0.86 (Mollayeva et al., 2016).

#### 2.6.5 Swedish national board of health and welfare physical activity questionnaire (BHW PA)

The BHW PA questionnaire comprises two separate questions about everyday physical activity and exercise. The BHW PA questions were designed to assess the level of PA (Olsson et al., 2016; The Swedish National Board of Health and Welfare, 2023) and identify those who do not meet the recommendations of 150 minutes of moderate activity per week (WHO, 2010; Bull et al., 2020). The two questions are combined to a total physical activity (PA) score that ranges from 3 to 19, where a greater score reflects a higher level of total PA. A value of 11 or more is the cut-off score for being sufficiently physically active. The BHW PA questions have shown to be valid in comparison with other self-assessment tools (Hagströmer et al., 2021) and are relatively valid in comparison with objective methods such as accelerometers (*r* = 0.31) (Olsson et al., 2016).

#### 2.6.6 Stroke Impact Scale (SIS 3.0)

SIS 3.0 is a stroke-specific self-assessment tool designed to identify and assess the consequences of a stroke (Duncan et al., 2003). It includes 59 items assessing 8 different domains of disability: strength, memory and thinking, emotion, communication, (instrumental) activities of daily living (ADL/IADL), mobility, hand function, and participation. All items in each of the 8 domains are evaluated on a scale ranging from 1 to 5. For each domain, a score ranging from 0 to 100 is calculated using the following equation: domain score = ((mean item score – 1) / 4) x 100 (Mulder et al., 2016), where a greater score reflects a more favorable outcome. In addition, a visual analogue scale (VAS) is used to assess self-rated recovery from 0 to 100, where 100 represents full recovery. The SIS 3.0 has been shown to be valid with a correlation coefficient from 0.44 to 0.84 compared with other self-assessment tools, and reliable with an ICC from 0.57 to 0.92 (Duncan et al., 1999). It also has good internal consistency with a Cronbach’s alfa from 0.86 to 0.95 (Jenkinson et al., 2013).

### 2.7 Data quality control

All data were imported to the IBM SPSS Statistics v 28 to create a study-specific database. A quality control process of the database was performed by two of the authors (MK and HMN). A validation of the data set was then performed by checking for logical consistency and completeness against the self-assessment tools. There were a few missing values in SIS, GDS and LiSat-11 and these were imputed according to decisions made by the research group; a total of 63 values were imputed amounting to less than 1% of all values in these three self-assessment tools. When the quality control was completed, the database was locked.

### 2.8 Statistical analysis

Sociodemographics, stroke characteristics and self-rated degree of recovery are presented by means of descriptive statistics. The comparison between participants and non-participants regarding gender and age was analyzed with the Mann-Whitney U test. All statistical analysis was performed using the IBM SPSS Statistics v 28 and *p*-values less than 0.05 were considered statistically significant.

## 3 Results

### 3.1 Participants and non-participants

Data for the participants (*N* = 160) and non-participants (*N* = 141) are presented in Table 2. A majority of the participants (*n* = 86; 54%) were men and the mean and median age was 73 years (SD±11) and 74 years (range 30– 91), respectively. There were no significant differences between the participants and non-participants regarding gender (*p* = 0.40) and age (*p* = 0.45).

**Table 2:** Comparison between the participants and non-participants in the Life After Stroke In Northern Sweden Study (LASINS)

	Participants	Non-participants	Significance
	(*N*= 160)	(*N*= 141)	test
Gender			*p* = 0.40
Men, *n* (%)	86 (54)	69 (49)
Women, *n* (%)	74 (46)	72 (51)
Age total groups (years)	73±11; 74, 30– 91	71±14; 74, 27– 98	*p* = 0.45
Men (years)	73±11; 74, 47– 91	71±13; 74, 38– 93
Women (years)	72±11; 74, 30– 91	71±15; 75, 27– 98

Data are presented as mean±SD; median, min-max. Comparison between participants and non-participants regarding gender and age were analyzed with the Mann-Whitney U test.

### 3.2 Sociodemographics, stroke characteristics and self-rated degree of recovery

Sociodemographics and stroke characteristics of the 160 participants are presented in Table 3. The majority of the participants (*n* = 134; 84%) were not working and two-thirds (*n* = 100; 62.5%) were cohabitant. About one third (*n* = 47; 29%) used some form of mobility device and a small proportion (*n* = 26; 16%) needed home help.

**Table 3:** Sociodemographics and stroke characteristics of the 160 participants in the Life After Stroke In Northern Sweden Study (LASINS)

	Total	Men	Women
	(*N*= 160; 100%)	(*n* = 86; 54%)	(*n* = 74; 46%)
Marital status
Living alone	60 (37.5)	23 (27)	37 (50)
Cohabitant	100 (62.5)	63 (73)	37 (50)
Vocational situation
Working	26 (16)	14 (16)	12 (16)
Disability pension	3 (2)	1 (1)	2 (3)
Old age retirement	131 (82)	71 (83)	60 (81)
Use of mobility device
Walking aids	39 (24)	16 (19)	23 (31)
Wheelchair	8 (5)	7 (8)	1 (1)
Home help	26 (16)	13 (15)	13 (18)
Time since stroke (months)	35±11; 34, 18– 61	35±11; 33, 18– 61	36±12; 38, 18– 60
Type of stroke
Ischemia	139 (87)	76 (88)	63 (85)
Intracerebral hemorrhage	21 (13)	10 (12)	11 (15)
Location of stroke
Right hemisphere	53 (33)	26 (30)	27 (37)
Left hemisphere	59 (37)	30 (35)	29 (39)
Cerebellum	13 (8)	9 (11)	4 (5)
Brain stem	3 (2)	1 (1)	2 (3)
Other*	32 (20)	20 (23)	12 (16)
Treatment
Thrombolysis	33 (21)	20 (23)	13 (18)
Thrombectomy	3 (2)	2 (2)	1 (1.4)
First time stroke	136 (85)	74 (86)	62 (84)
Co-morbidity	143 (89)	76 (88)	67 (91)

Data are presented as *n* (%) and mean±SD; median, min-max. *Other = stroke localized in both hemispheres or unidentified.

The mean time since stroke onset was 35 months (SD±11, range 18– 61) and the most common type of stroke was ischemic (*n* = 139; 87%). A large proportion of the participants had a left or right hemisphere stroke (*n* = 112; 70%) and about one quarter (*n* = 36; 23%) had received thrombolysis and/or thrombectomy. A majority (*n* = 136; 85%) had a first-time stroke and some co-morbidity (*n* = 143; 89%), most commonly hypertension and type 2 diabetes, and atrial fibrillation and hyperlipidemia.

**Fig. 2:**
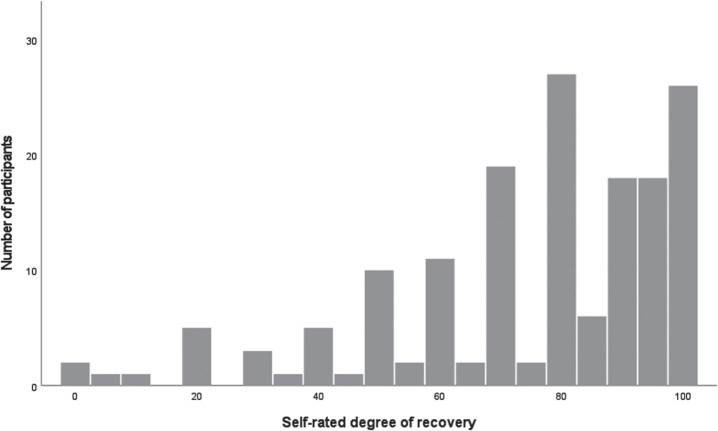
Self-rated degree of recovery (Stroke Impact Scale, SIS 3.0) among 86 men and 74 women in the Life After Stroke In Northern Sweden Study (LASINS).

The 160 participants’ self-rated degree of recovery is presented in Fig. 2. The mean and median value was 75 (SD±24) and 80 (range 0– 100), respectively. A total of 97 participants (61%) rated 75 or higher and 24 (15%) rated themselves to be fully recovered (i.e., 100 on the VAS). The mean and median value for the men was 72 (SD±26) and 80 (range 0– 100), respectively, and for the women 78 (SD±20) and 80 (range 0– 100), respectively; there were no significant differences (*p* = 0.50) between the men and women.

## 4 Discussion

The overall objective of the LASINS is to provide an in-depth understanding of factors of importance for a healthy and successful life after stroke. Our focus is on sleep disturbances, depressive symptoms, fatigue and physical activity and the interaction with remaining impairments, activity limitations, participation restrictions and life satisfaction. The data collected in the LASINS will allow us in forthcoming studies to address life after stroke from several perspectives.

Ischemic stroke was the most common type among the participants, which is consistent with the occurrence of stroke worldwide (Saini et al., 2021). The gender distribution is identical to the Swedish stroke population (Dahl et al., 2020; Riksstroke, 2022a) and the participants mean age is also very similar to the Swedish stroke population (Riksstroke, 2022a) as well as internationally (Yousufuddin et al., 2017). More men were cohabitant which is also consistent with previous national and international studies; women who suffer a stroke are more likely to live alone or be widowed as they acquire their stroke later in life (Eriksson et al., 2021; Petrea et al., 2009).

Studies of remaining impairments and physical activity have generally been performed in the early phase after stroke. Therefore, we wanted to include people at least one year after stroke onset. In the LASINS the participants are from 18 months post stroke to just over six years, with a mean close to three years. This enable us to assess people who are in a stable, and thereby chronic phase after stroke and also focus on the long-term perspective among stroke survivors.

As expected, most of the participants had some form of co-morbidity, with hypertension and type 2 diabetes being the most common. This corresponds to the most common co-morbidities in the Swedish stroke population (Appelros et al., 2021) and are in agreement with the global stroke population (Johnson et al., 2019).

More than half of the participants rated their degree of recovery to 75 out of 100 and 15% rated themselves to be fully recovered. This is in agreement with our data that only a minority of the participants were using a mobility device and not needing any home help (cf. Table 3). A similar pattern of recovery has been described in the Swedish stroke population (Riksstroke, 2022a). At a one-year follow-up, 80% of stroke survivors under the age of 75 years described that they had recovered to the extent that they were walking independently without a mobility device and only one fifth had a need for help in their home (Jönsson et al., 2014; Riksstroke, 2022b). However, among the participants in the LASINS there were those who rated that they had recovered only to a limited degree or not recovered at all. Thus, the population included in the LASINS represent all degrees of recovery, yet the majority can be considered to have had a mild to moderate stroke.

We made great efforts to obtain as many participants as possible, including sending one reminder. This resulted in a response rate of 53% of those matching the inclusion criteria (cf. Fig. 1). As we wanted to include those who had a confirmed diagnosis of stroke, and were able to understand and answer a questionnaire and the self-assessment tools in Swedish, a fairly large proportion were excluded. Among the excluded where those with a TIA together with 25% of those treated in the stroke unit that had deceased. This emphasizes the diversity of stroke as well as the severity of the disease. When comparing the participants and the non-participants, there was no significant difference regarding their age and gender. Thus, our sample is likely to represent the population of stroke survivors in northern Sweden.

Data in the LASINS were collected using several generic and stroke specific self-assessment tools that were selected based on several criteria. The tools in the LASINS are valid and reliable, and cover the main components of the ICF (WHO, 2023). As the self-assessment tools contain a relatively large number of questions this can lead to a risk of selection bias because of the potential burden of answering as well as missing data. There were, however, only a very small number of missing values in three of the six self-assessment tools (less than 1%), and with the relatively large number of participants, fairly detailed inferences can be made from multivariate analysis in forthcoming studies.

Although the LASINS will provide an in-depth description of life after stroke there are some study limitations. The data collected will be used to describe the current life situation of stroke survivors, with a specific focus on sleep disturbances, depressive symptoms, fatigue and physical activity. However, due to the cross-sectional design, causal inferences cannot be made, so follow-up data are required. As a majority of the participants rated themselves to be fairly well recovered, our results and inferences should be limited primarily to those with a mild to moderate disability. Even though the six self-assessment tools cover various aspects of health and well-being, the quantitative design limits our ability to explore long-term outcome from a truly person-centered perspective. In the future, qualitative research studies will therefore be conducted. Qualitative research deals with experiences, perceptions, and meaning, thereby allowing an understanding of a person’s inner perspective and complementing the quantitative data.

## 5 Conclusions

The LASINS is a cross-sectional, population-based survey with a neurorehabilitation approach and these baseline data together with forthcoming studies will describe stroke-related impairments, activity limitations, participation restrictions and life satisfaction more than one year after stroke, and deepen our understanding of factors of importance for a healthy and successful life after stroke. Given the global prevalence of adults with stroke, the LASINS will provide knowledge of clinical and scientific as well as societal relevance and is expected to be a foundation to create targeted interventions to improve rehabilitation for people after stroke.

## Conflict of interest

None to report.

## Author contributions

MK, HMN and JL conceptualized the study. MK and HMN performed the data collection and analysis. MK, HMN and JL drafted the original version of the manuscript, reviewed and edited the revised versions of manuscript and subsequently approved the final version. MK and HMN contributed equally to this work and thereby share first authorship.

## Data availability

All data are archived according to the Swedish Act concerning the Ethical Review of Research Involving Humans and are available from the authors upon request.
